# Are Linguistic Prediction Deficits Characteristic of Adults with Dyslexia?

**DOI:** 10.3390/brainsci11010059

**Published:** 2021-01-06

**Authors:** Paul E. Engelhardt, Michelle K. Y. Yuen, Elise A. Kenning, Luna Filipovic

**Affiliations:** 1 School of Psychology, University of East Anglia, Norwich NR7 7TJ, UK; e.kenning@uea.ac.uk; 2 Department of Psychology, University of Bath, Bath BA2 7AY, UK; michelleyuenky@gmail.com; 3 School of Politics, Philosophy, Language, and Communication Studies, University of East Anglia, Norwich NR4 7TJ, UK; l.filipovic@uea.ac.uk

**Keywords:** dyslexia, reading disability, sentence processing, linguistic prediction, cloze probability, semantic plausibility

## Abstract

Individuals with dyslexia show deficits in phonological abilities, rapid automatized naming, short-term/working memory, processing speed, and some aspects of sensory and visual processing. There is currently one report in the literature that individuals with dyslexia also show impairments in linguistic prediction. The current study sought to investigate prediction in language processing in dyslexia. Forty-one adults with dyslexia and 43 typically-developing controls participated. In the experiment, participants made speeded-acceptability judgements in sentences with word final cloze manipulations. The final word was a high-cloze probability word, a low-cloze probability word, or a semantically anomalous word. Reaction time from the onset of the final word to participants’ response was recorded. Results indicated that individuals with dyslexia showed longer reaction times, and crucially, they showed clear differences from controls in low predictability sentences, which is consistent with deficits in linguistic prediction. Conclusions focus on the mechanism supporting prediction in language comprehension and possible reasons why individuals with dyslexia show less prediction.

## 1. Are Linguistic Prediction Deficits Characteristic of Adults with Dyslexia?

Dyslexia is a learning disability of genetic origin that affects an individual’s reading attainment, despite adequate intelligence and opportunities to learn [1]. Epidemiological estimates place the incidence rate at approximately 5–10% of the population and characteristic features of dyslexia are difficulties in phonological abilities, rapid automatized naming, short-term/working memory, and processing speed. These issues have been encompassed within several “multi-factorial” deficit theories of developmental disorders (e.g., [2,3]). The current study sought to identify whether deficits in linguistic prediction are also characteristic of individuals with dyslexia. There is currently only one study in the literature (i.e., [4,5]), which has shown differences between individuals with dyslexia and typically-developing controls in terms of prediction in language comprehension. Deficits in prediction may also be related to the fact that individuals with dyslexia are known to rely more heavily on context when reading in order to overcome (or partially compensate) for slow word decoding [6,7].

## 2. Prediction in Language Comprehension

It has been shown that more predictable words are read faster [8,9], and predictability is one of the “big three” variables that affect fixation durations in reading [10]. Prediction in language processing is often argued to facilitate communication efficiency [11,12,13,14,15,16,17]. However, the claim is not without controversy, as some have suggested that predictions are often wrong (given the large number of possible continuations of any given input string), and second, prediction has the potential to result in excessive cognitive load in the event that predictions do turn out to be wrong [18,19]. However, despite this controversy, there is a large event-related potential (ERP) literature showing an increased N400 amplitude when listeners/readers encounter unexpected or unpredictable words (e.g., [20,21,22,23]).

Before going into the ERP results in detail, it is important to define what we mean by “prediction”. Typically, in psycholinguistics, prediction is referred to as the comprehension system being able to “predict” an upcoming linguistic unit (e.g., a word) ahead of the bottom-up input. It is also referred to as “anticipation” or “expectation”, and we do not differentiate between these different labels. Some more recent proposals have suggested that instead of prediction, “preparedness” is a better way to conceive of the systems’ head start on upcoming material. Another important topic, which is related to prediction is “integration”, and the relationship between integration and prediction [24,25,26]. For extensive discussions of these issues, we recommend review papers by Kuperberg and Jaeger [27] and Ferreira and Chantavarin [28]. However, there is very clear evidence that comprehension system operates incrementally and derives semantic and syntactic interpretations, as each word in a sentence is encountered [29]. Integration is the process of combining a new word with the partial syntactic structure, which results in an interpretation of the input up to that point, leading to propositional meaning. It is important to note that this process takes into account semantic integration and schema/situational factors, as well as syntactic factors [30]. In our conceptualization, we view facilitated integration effects and prediction as two sides of the same coin (see also [31]). If a comprehender is predicting something in advance of bottom-up input, then the integration of that word into the sentence context will be faster, as bottom up information confirms the prediction.

Prediction in language comprehension has been most often operationalized using cloze probability [32]. Cloze probabilities typically range from 1.0 to 0.10. These values are calculated by having a group of participants complete a sentence fragment (i.e., a sentence with a missing word) with the word they feel is the best or most natural fit for that sentence. A cloze probability of 1.0 means that all participants provide the same final word for the sentence, which is also called a singleton response. As the cloze probability of a sentence decreases, there is less predictability in the sentence fragment, and as a result, more words “fit”. If 100 participants are given *The hunter shot and killed a large _________.*, and 36 of them write *deer,* then the cloze probability of the sentence is 0.36 (i.e., the most frequently provided response from the group of participants). Likewise, comprehenders will be faster to read/process the sentence when the final word is *deer*, as compared to a lower probability completion (e.g., *bear*). Finally, the sentence stem *The hunter shot and killed...* leads to less prediction (or is less predictable) compared to a sentence that has a higher cloze probability (for examples see Table 1).

Prediction can be experimentally assessed using differences in cloze probability in one of two ways. The first is like that described in the previous paragraph. The same sentence stem can be presented to participants with different final words (e.g., *deer* vs. *bear*). The alternative is to use the same final word (e.g., *deer*) and to present it in two different sentence contexts [33]. One of which would make the final word more predictable as compared to the other. Both of these have been employed in prior ERP studies. Either way, if a word is less predictable (or more unexpected), then it will result in longer processing time or in a greater N400 amplitude, see also [5]. The N400 component has been most often associated with semantic-integration issues in typically-developing adults [26,27,34,35], but there is some N400-type ERP work more specifically related to phonological processing in dyslexia [36,37]. The original demonstration of the N400 was shown for sentences containing an anomalous (or implausible word), which resulted in a negative going potential beginning 200–300 ms after the onset of the anomalous word and peaking at approximately 400 ms [38]. An attenuated N400 has also been observed from plausible but unexpected words [39], and critically, lower cloze probability words result in larger N400s [35]. Thus, in sentence processing, the N400 is widely viewed to result from a combinatorial semantic integration process in which words are integrated with prior context [34].

A second view suggests that the N400 also reflects difficulty of lexical access in long-term memory. By this explanation, contextual information allows certain words to be accessed and retrieved from memory more easily [22,23]. In highly constraining contexts, the comprehension system may be able to make very specific predictions about particular lexical items, in which case the comprehension system can get a head start on identification and access. The N400 in these ‘‘predictable’’ cases is reduced. Thus, the language comprehension system is sensitive to the relationships between words in a sentence and what is likely to follow next, and it is also sensitive to variations in cloze probability, which are measurable within 300 ms of word onset. Priming studies have also identified that the amplitude of the N400 is influenced by many sub-lexical, lexical, and post-lexical processes [40].

To summarize, most recent theoretical proposals suggest a relationship between prediction and integration, e.g., [27,28,31,41]. Words that are predicted and/or predictable can be more easily integrated because the processing system can get a head-start, likely through (semantic) pre-activation or access in long-term memory. We do not view these assertions as opposed to preparedness, but instead, as describing different points along a “predictability” continuum. The mechanism, which supports this, is gradient probabilistic semantic activation. This mechanism was most clearly explicated by Staub et al. [42], who proposed a “race-to-threshold” model, primarily designed to account for the reaction times in a production version of the cloze task, but which also has clear implications for reaction times in comprehension versions as well [43]. The production version of the task requires participants to produce a word following a written or spoken sentence fragment, and the comprehension version requires a response based on whether the final word of a complete sentence makes sense or not. In both versions, there is accrual of semantic activation in part of the semantic network. This activation is based on multidimensional relations between words in the sentence context. In addition, the “race” nature of the model describes the fact that multiple lexical candidates are activated in parallel and probabilistically, rather than single candidates serially.

### 2.1. Relationship between Prediction and Language Abilities

Linguistic prediction in dyslexia was first studied by Huettig and Brouwer [4]. However, Huettig and colleagues [44,45,46] have also investigated linguistic prediction based on literacy level, see also [47]. Their results revealed that compared to literate individuals, illiterate individuals were less likely to show anticipatory eye movements (i.e., show less linguistic prediction). Participants were asked to listen to a sentence, while viewing an array of objects. An example was, *Now you are going to see a tall door,* the visual array contained the target (*door*) and three distractors (*button*, *flower,* and *drum*). The sentences were designed to semantically constrain the likely noun given the adjective. Upon hearing the adjective, the high literacy group shifted their eye gaze to the target. In contrast, the low literacy group shifted their gaze only after hearing the noun, resulting in a 1 s delay in shifts of attention. Huettig and colleagues concluded that increased reading experience results in a greater ability to make predictive eye movements when mapping spoken language onto the external environment. These findings were supported in a more recent ERP study [5], which showed that comprehenders with poorer literacy were less likely to engage in predictive processing. Perhaps more interestingly, the differences based on literacy actually emerged in an earlier time window (170–300 ms) than would be expected for a “typical” N400. The scalp distribution of the early negativity was more anterior, and thus, similar to a component referred to as Phonological Mapping Negativity [48].

Returning to the issue of prediction in dyslexia, Huettig and Brouwer [4] hypothesized that individuals with less reading ability might also show deficits (or less fine-tuning) of language-mediated anticipatory eye movements. They conducted two experiments to examine anticipatory eye movements in adults with dyslexia. In Experiment 1, participants heard a target word (*beaker*) in the context of an array that contained four objects. Three of the objects were related to the target and one was a control object. The related objects were “shape”, “semantic”, and “phonological” competitors. One object had a similar shape as the target, one was semantically related to the target, and one shared the same consonantal onset and vowel nucleus (i.e., a phonological competitor). Looks to the phonological competitor emerged earlier than the other two, but crucially, there was no difference between controls and individuals with dyslexia, suggesting that individuals with dyslexia process spoken language and direct attention in a similar way and with a similar time course as individuals without reading difficulties.

In Experiment 2, participants heard instructions (e.g., *look at the displayed piano*), while viewing four object arrays. The determiner was gender marked (in Dutch), and only agreed with the target object. Thus, participants could use gender information to predict the target. Results showed that both groups anticipated the target, but there were significant differences between groups. Controls showed anticipatory eye movement 400–800 ms after the onset of the determiner. Individuals with dyslexia showed anticipatory eye movements 1400–1500 ms after the onset of the determiner. Thus, individuals with dyslexia showed a 1 s delay compared to controls, suggesting that individuals with dyslexia are not as efficient at linguistic prediction.

### 2.2. Multiple Deficit Theories of Dyslexia

Dyslexia is associated with several neurocognitive deficits (e.g., phonological abilities, processing speed, rapid automatized naming, etc.). There is more evidence for some deficits (e.g., phonological abilities) compared to others (e.g., working memory). The primary purpose of the current study was to confirm another suspected deficit (i.e., linguistic prediction), which to date has only been shown in a single study [4,5]. Dyslexia has been traditionally defined via a discrepancy criterion, in which reading ability falls 1.5–2 SDs lower than general intelligence and non-verbal cognitive ability [49]. Theoretical models of the disorder (e.g., the Double-Deficit theory) have generally aligned with the discrepancy notion [50,51], and focused on characteristics directly linked with reading (e.g., phonological abilities). However, more recently, models of developmental disorders have tended to shift to multiple-deficit (type) theories [2,3,52,53], and as such, can account for wider ranging and more complex cognitive phenotypes, as well as capturing the inter-relations between various deficiencies. It is also important to acknowledge that dyslexia is not strictly associated with cognitive impairment. Recent work has investigated sensory [54,55], proprioceptive [56], and sensorimotor [57,58] impairments. At present, we do not know whether deficits in linguistic prediction are causally linked to the other known characteristics of dyslexia, and that is not a major goal of the current study. Instead, the goal of the current study was to provide additional confirmatory evidence for deficits in linguistic prediction in dyslexia.

### 2.3. Current Study

The current study investigated linguistic prediction in dyslexia. Like Huettig and colleagues, we thought spoken language was the best modality to assess prediction, given that dyslexia is defined by impairments in reading. Huettig and Brouwer [4] showed that controls and individuals with dyslexia were able to make language-mediated anticipatory eye movements, but dyslexics were substantially slower than controls. In the current study, we investigated prediction in dyslexia using a version of the cloze task, in which participants made speeded-plausibility judgements [59]. There were several reasons why we chose this task, but the main one concerns a potential weakness in Huettig and Brouwer [4]. Specifically, their task focused on only one aspect of syntactic agreement (i.e., prediction of a noun based on a gender marked determiner). It is currently unknown whether individuals with dyslexia experience similar delays in prediction across other syntactic structures and with other predictive cues. Thus, we sought to assess prediction in dyslexia in a wider range of linguistic contexts, which ensures greater/better generalizability of findings.

As mentioned above, the cloze task is one of the most widely used measures to assess linguistic prediction in sentences contexts [32]. In the traditional form of the task, participants are asked to produce a written continuation for an incomplete sentence. Averaging responses from many participants provides the mean cloze probabilities for individual words provided. Thus, cloze probability is an objective measure of a word’s predictability. We utilized published cloze norms for British English [60], and we manipulated the final word of each sentence (see Table 1). Two semantically “plausible” continuations were taken from the published norms. We used the word with the highest cloze probability for each item for the “high” continuation condition. For the “low” continuation condition, we utilized a word with the lowest (or near to lowest) mean cloze probability for each item. This creates the largest difference in terms of the most predictable word to the least predictable word. Finally, for an “anomalous” continuation condition, we chose a word that was semantically anomalous for the sentence context.

The mean cloze probabilities of the items in Arcuri et al. [60] ranged from 0.09 to 1.0. At the upper end (i.e., 1.0), sentences are so constraining that all participants produce the same word (i.e., a “singleton” response). At the low end (i.e., 0.09), the highest probability word was given by only 9% of participants. So, as the cloze probability increases from low to high, the distribution of responses changes. Low constraint sentences, by definition, have many different words that fit the context reasonably well. High constraint sentences, in contrast, have very few words that fit the context, and so, the range of words naturally narrows. We refer to this range of cloze probability in terms of the “sentence constraint”. Following Staub et al. [42], we divided the experimental items into two categories (high-and low-constraint), based on the mean cloze probability of the highest probability response in the published norms. Items less than 0.50 were defined as “low” constraint, and items greater than 0.50 were defined as “high” constraint. Finally, we tested individuals diagnosed with dyslexia and compared them to individuals with no history of reading difficulties.

We predicted that high-constraint sentences would be processed faster than low-constraint sentences. We also expected differences between the high-and low-continuations of each sentence. The anomalous items were included because they require a “no” does not make sense response, and were treated as filler items (i.e., they were not included in the main statistical analyses). However, the difference between high-and low-continuations should be greater in high-constraint sentences than in low-constraint sentences. With respect to dyslexia, we expected overall slower reaction times, given reports of delayed linguistic prediction, but also generally slower processing speed [61,62,63]. However, if individuals with dyslexia do not predict as much as controls then the difference between the high-and low-continuations would be significantly less pronounced, and possibly, not significantly different.

## 3. Method

### 3.1. Participants

Forty-one adults with dyslexia were recruited and 43 undergraduate psychology students were tested as typically-developing controls. Both groups were recruited from the campus of the University of East Anglia (UEA). Approval for research protocols was given by the “University of East Anglia, School of Psychology, Research Ethics Committee” (Date approved: 04-12-2017; Ref number: 2017-0540-000804). The mean age of dyslexics was 21.7 years (SD = 2.67) and 41.5% were male. The mean age of controls was 19.7 years (SD = 1.74) and 14% were male. All participants with dyslexia verified that they had a positive diagnostic assessment for dyslexia by a qualified professional in the past and were on the disability register at UEA (for learning disability—dyslexia). In addition, we had all participants complete a RAN letters (dyslexics = 18.09 s (5.37); controls = 14.83 (4.82)) and RAN numbers (dyslexics = 17.11 s (5.70); controls = 13.10 (2.50)) task. The means for these two tasks were significantly different between groups (letters: *t*(82) = −2.94, *p* < 0.01 and numbers: *t*(82) = −4.21, *p* < 0.001), which is highly similar to prior dyslexia samples drawn from the same population, see also [64,65,66]. All participants were native speakers of British English with normal or corrected-to-normal vision and hearing. Controls were compensated with psychology pool participation credits, and dyslexics with psychology pool participation credits or £5.

### 3.2. Rapid Automatised Naming

All participants completed both a letter and a number RAN test using the Comprehensive Test of Phonological Processing (CTOPP 2). The RAN task requires participants to name a series of letters or numbers sequentially out loud as quickly and accurately as possible. The time taken to complete an array was recorded with a stopwatch. Participants completed one letter and one number array for practice, and two served as the critical trials (i.e., one letter array and one number array). The score for each task was the total time that was needed to complete the task, higher scores indicate worse performance. Each array consisted of four rows of nine items. Letters and numbers were presented in Arial font, and all items appeared on the same side of white A4 paper. The standardised procedures of administration for this task were followed as described in the test manual. 

### 3.3. Linguistic Prediction Task

The 108 experimental items and three practice items were obtained from Arcuri et al. [60]. Each sentence was recorded three times with a different continuation. The first had the highest (cloze probability) completion. The second had the lowest (or second to lowest) cloze probability completion, and the final one, had an anomalous completion (i.e., the sentence did not make sense). We ensured that the initial phoneme of each of the final words were not the same. If the lowest probability completion had the same initial phoneme as the high probability completion, we used the next lowest completion. Because the final word was different for each of the three completions, we carefully scrutinized the final words for various lexical variables (see Table 2). Results of those analyses showed that word length and phonological neighborhood size of the three different completions were significantly different, but the frequency (assessed through various measures) was not significantly different. Likewise, there were no differences in concreteness, imagability, or phonological neighborhood frequency. For the critical high-and low-continuations, there were significant differences in the length (number of characters: *t*(108) = −3.58, *p* < 0.01, and number of syllables: *t*(108) = −2.59, *p* < 0.05), as well as phonological neighborhood size *t*(108) = 2.62, *p* < 0.05. The three versions were placed into different lists and rotated in a Latin square design. 

### 3.4. Apparatus

The experiment was programmed with the Experiment Builder experimental software, and run on a laptop PC. Participants listened to the sentences either from the computer speakers or through headphones depending on the testing location.

### 3.5. Design and Procedure

The design was a 2 × 2 × 2 (Group: dyslexia vs. control × Constraint: high vs. low × Continuation: high vs. low) mixed model, in which constraint and continuation were within subject and group was between subject. Recall that the anomalous items were considered fillers in part because that condition required a different response (two conditions required a “yes”—makes sense response and one condition required a “no”—does not make sense response). Mean RTs (SDs) for the anomalous items were: control-low constraint = 1213 ms (305), control-high constraint = 1153 ms (283), dyslexic-low constraint = 1520 ms (450), dyslexic-high constraint = 1500 ms (444) (see also Supplementary Materials Section B). Participants completed three practice trials, and 108 experimental trials (36 in each of the three conditions). Trials were presented in a random order for each participant.

Participants first completed a short demographic questionnaire and then did the RAN task. In the linguistic prediction task, participants were instructed that they would hear a sentence. Their task was to indicate whether the sentence made sense, and that it was important that they responded as quickly as possible, consistent with making as few errors as possible. If the sentence made sense, they should press “M”. If the sentence did not make sense, they should press “C”. If they were unsure, they could press the “spacebar” to indicate a non-response. Each trial began with a drift correct dot, which indicated that the participant could press the spacebar to initiate the trial. A fixation cross appeared in the center of the screen, and after 1000 ms, the sentence was played. After participants responded, the next trial began. The reaction time was measured from the onset of the final word to when the participant made their response. Most participants were tested in a quiet environment (i.e., laboratory cubicle) in which case they listened to sentences through the computer speakers. A few participants were tested in other rooms on campus, and in this case, they listened to sentences through headphones. The entire experimental session took approximately 20 min.

### 3.6. Data Screening and Analysis

There were 822 incorrect responses, and 71 non-responses. (Two items showed many incorrect responses, and the reaction times from the correct responses were also elevated. We elected to remove these two items from all analyses.) These trials were excluded from all analyses and constituted 9.7% of the data. Second, the reaction time distributions were examined to exclude high and low outliers. We utilized 200 ms as the threshold for low values and 5000 ms for high values. There were 37 trials outside these thresholds and were excluded. Thus, in total, we excluded approximately 10% of the data. 

The reaction times were submitted to a Linear Mixed Effects model using R [67] and *lmer* [68]. Results include *p*-value estimates from the lmerTest package. Fixed effects for continuation, constraint, and group were included. The random effects structure was maximally specified with random intercepts for participants and items [69]. In the event of convergence problems, the model was simplified until convergence was satisfied. 

## 4. Results

### 4.1. Main Analysis

**Figure 1:**
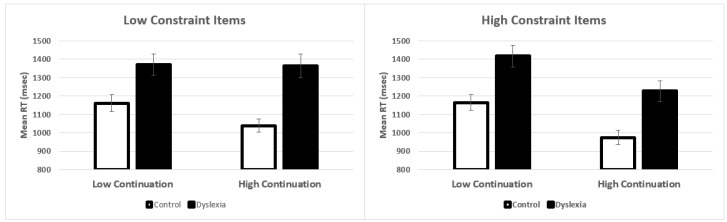
Mean reaction times. (**Left panel**) shows the results for the low-constraint items and the (**right panel**) shows the high-constraint items. Error bars show the standard error of the mean.

The main effect of Group was significant (see Table 3), in which individuals with dyslexia were slower than controls. The 3-way interaction between Group × Constraint × Continuation was also significant. (The full model results and code are provided in the Supplementary Materials (see Section A). The effect sizes for the key results, that is, the main effect of group and the three-way interaction were 0.152 and 0.056, respectively. These effect sizes are partial eta squared calculated based on by-subject means.) The means are presented in Figure 1. The significant three-way interaction was followed up by multiple comparisons using Tukey contrasts (see Table 4). In low-constraint items, three paired comparisons were significant and one was not. Controls were significantly faster than individuals with dyslexia, and controls were faster with high compared to low continuations. However, individuals with dyslexia showed no significant difference between the high-and low-continuations, indicating that they do not predict as much in low-constraint items. Results revealed that all of the paired comparisons for the high-constraint items were significant, suggesting significant differences between the high-and low-continuations, and that controls were significantly faster than individuals with dyslexia. As one further analysis, we compared the high vs. low constraint items. Results (additional contrasts in Table 4) showed that both groups were not faster with the low-continuations but were significantly faster with the high-continuations, suggesting that participants were faster to respond “yes” in high-constraint items than in low-constraint items with high-continuations.

### 4.2. Age and Gender

**Figure 2:**
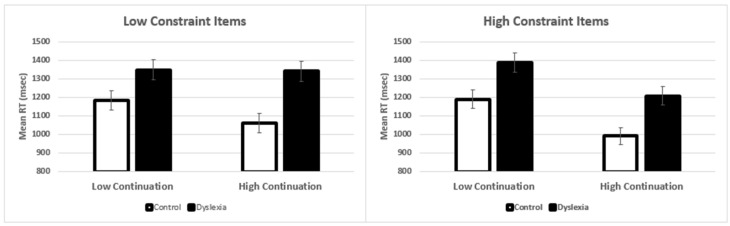
Gender adjusted mean reaction times. Left panel shows the results for the low-constraint items and the right panel shows the high-constraint items. Error bars show the standard error of the mean.

Our groups differed in age and gender, and in order to ensure that the group differences reported above were not due age and gender, we conducted additional analyses. When age was included in the statistical model, it was not significant (*p* = 0.40) and it did not interact with any of the other three variables. In contrast, when gender was included in the model, it did produce a significant main effect (*p* = 0.042), but importantly, gender did not interact with any of the other three variables. The inclusion of gender did not affect the main pattern of findings that were reported in the prior section (Table 3 and Table 4). However, there was a dissociation between age and gender, in that when age was included in the model the main effect of dyslexia and the three-way interaction were stronger (i.e., produced larger effects), whereas slightly smaller effects occurred when gender was included. In Figure 2, we have presented the adjusted means when controlling for gender, and as can be seen, there is very little change to the overall patterns. Therefore, we are confident that effects reported in the main analysis section are not due to age or gender differences in the samples.

## 5. Discussion

To summarize the main findings of this study, we found that individuals with dyslexia showed clear differences from controls in terms of prediction in low-constraint items. This was evident in the non-significant paired comparison between the high- and low-continuations within low-constraint sentences. In contrast, controls showed significant differences between the high- and low-continuations, in both the highly predictable and less predictable items. There was also a significant group difference in which individuals with dyslexia were slower compared to controls. In addition, we also ensured that differences were not due to age or gender. There are two important take home messages from this study.

The first is that individuals with dyslexia showed no evidence of linguistic prediction in sentence contexts which afforded less prediction (see also scatterplots in Supplementary Materials, Section B). The scatterplots show that in controls the lines of best fit are nearly parallel for the high and low continuations, and there is clear separation between them. In contrast, in dyslexics, something quite different happens, in particular, with the low continuation items (i.e., the line of best fit is slightly positive). As far as we are aware, this is the only data point in the literature to actually show higher reaction times for higher cloze probability items [42]. We are not in a position to definitely say why this occurred, but it is in all likelihood due less incremental-semantic processing (i.e., shallower processing of the sentence until the final word is encountered). Whether this is due to impairment/reduction in working memory remains an open issue. What is clear is that there is no separation between the high and low continuations at the lower end of the cloze probability continuum, which again, is a clear indication of less linguistic prediction.

The second take home message was that these findings demonstrate that linguistic prediction is reduced in individuals with dyslexia more generally than has been demonstrated previously. In our stimuli, the manipulated sentence-final words occurred in a wide variety of syntactic structures. In some items, the final words were direct or indirect objects, and in other cases, they were simply adjuncts. This variety ensures that our materials are more generalizable to within sentence prediction effects. We noted a potential generalizability issue in Huettig and Brouwer [4], as that study investigated prediction from a gender marked determiner, and because it was a Visual World study, the sentence structures were essentially all the same, and in effect, only served as carrier phrases for the critical determiner and noun. In Huettig and Brouwer, the presence of an additional word (displayed) provided additional time for prediction to occur. Ultimately, however, the conclusions of both studies are the same, namely that adults with dyslexia showed reduced linguistic prediction compared to controls. Our results suggested that individuals with dyslexia cannot predict in low-constraint sentences, and Huettig and Brouwer showed that dyslexics can make predictions, but are substantially slower to do so, similar to what we observed in high-constraint sentences. 

There are two additional points worth highlighting. The first relates to general processing speed differences in dyslexia. Several theories of dyslexia suggest differences in general processing speed [61,62,63,70,71]. Huettig and Brouwer [4] argued that the 1000 ms latency in anticipatory eye movements could not be due to differences in processing speed, because their Experiment 1 did not show significant differences between groups. However, that experiment was essentially about the activation of different types of semantic knowledge in response to hearing a noun. The named word never appeared in critical displays, and as a result, that experiment was not about prediction per se. We do not see any faults in their line of reasoning. However, at the same time, we do feel that the issue of processing speed is likely more complicated than Huettig and Brouwer [4] suggested.

In the current study, we observed baseline differences in reaction time between controls and individuals with dyslexia (i.e., a main effect of group). Grand means from the critical conditions indicated that individuals with dyslexia were on average 260 ms slower to make their response compared to controls. However, these baseline differences do not affect our main conclusions because the main conclusions were based on the comparison of low vs. high continuations. At this juncture, we can safely conclude that baseline reaction time differences are likely (or partially) due to some general differences in processing speed, but at the same time, we can also say that individuals with dyslexia do not show evidence of prediction in low-constraint sentences. One obvious next step will be to conduct an ERP study to examine N400 modulations to the low- and high-continuations, and how the time course of those amplitude changes as a function of group (control vs. dyslexia).

The second main point worth highlighting concerns the mechanism underlying prediction and the Race-to-Threshold model proposed by Staub et al. [42]. Recall that Staub et al.’s model was based on an experiment in which participants produced the final word rather than made semantic-acceptability judgements. One of the main arguments made by Staub et al. was that the reaction time differences were not due to semantic associations between the words in the sentence stem and the word produced by participants. In our study, participants did not produce the final word but had to process the final word with respect to fit with the rest of the sentence. Likewise, we examined the semantic relationships between individual words in the stem and the final words, and did not find semantic associations to be a strong driver of our reaction time findings. These results indicate that the accrual of semantic (pre-)activation is based on the combination of words in the sentence rather than semantic associations between individual words. This is the important data point which really shows that the task is about “prediction and integration” rather than simple semantic activation driven by association.

### 5.1. Limitations

We think that there are three main limitations to the study. The first is that our participants with dyslexia were all university students. As such, they likely represent an upper achieving (high literacy) sample of dyslexics, what some may refer to as “compensated” dyslexics. Further experiments testing community-recruited adults may show even greater differences in linguistic prediction than those reported here. The second limitation is that we did not have participants complete diagnostic assessments in order to be included in the study. Instead, we targeted our dyslexia recruitment particularly on individuals (verified by the research team to be) on the disability register at the university, and in order to be on the disability register, all students must provide evidence of a prior positive diagnosis of dyslexia by a qualified educational psychologist or a certified dyslexia-specialist education practitioner. The third limitation is that the high- and low-probability completions were significantly different in terms of length. We could not do anything about this difference as we relied on materials that came from a published corpus. There are several points to make about this limitation. The first is that length and frequency were definitely excluded as confounds in the reaction time results from Staub et al. The second is that length difference do not affect the group comparisons, as both dyslexics and controls experienced the same differences. Finally, the length difference, in absolute terms, was quite small (i.e., less than one character), and so, the significance of this difference is in some way affected by the high power of the design (i.e., 36 trials per condition). We also note that the phonological neighborhood size was significantly different between the high- and low-probability continuations. However, the direction of the difference should work in the opposite way to the expected reaction time differences between high and low continuations.

### 5.2. Conclusions

The findings from this study are largely consistent with the prior study that examined linguistic prediction in dyslexia. However, results extend the existing literature by demonstrating prediction deficits across a wider range of syntactic structures, which ensures that our findings are more generalizable to prediction in everyday language. In a recent paper, Huettig and Pickering [47] argued that prediction benefits are attributable to increased experience with written language, see also [5]. The basic idea is that increased exposure to written language fine-tunes lexical representations, specifically leading to stronger predictive contingencies between words in the lexicon. If these assertions are correct, then weaker linguistic prediction in dyslexia may be due to suboptimal reading experience and a tendency to avoid reading. The current results suggest that deficits in linguistic prediction (in adults with dyslexia) are likely restricted to situations in which the upcoming words are “less” predictable, that is, we observed no evidence of linguistic prediction in dyslexia in less constraining sentences. Despite these clear findings with respect to reduced linguistic prediction in dyslexia, at present, we do not know how deficits in linguistic prediction are related to other known characteristics of dyslexia, and in particular, whether they are due to cognitive or sensory/sensorimotor impairments. Future large-scale studies are needed to ascertain causal factors in prediction deficits, and also how prediction deficits may be accounted for within multi-factor models of the disorder.

## Figures and Tables

**Table 1:** Example stimuli, with the three types of continuation. Cloze probability in parentheses from Arcuri et al. [60].

	High	Low	Anomalous
Low Constraint			
The ruby was so big it looked like a ______.	Cherry (0.14)	Tomato (0.07)	Jacket
I don’t know why he didn’t take his ______.	Medicine (0.14)	Umbrella (0.09)	Pavement
They went to the rear of the long ________.	Queue (0.15)	Train (0.06)	Nails
Hank reached into his pocket to get the ___.	Money (0.30)	Change (0.09)	Shade
The hunter shot and killed a large _______.	Deer (0.36)	Lion (0.06)	Wind
High Constraint			
The sail got loose so they tightened the __.	Rope (0.54)	Mast (0.06)	Idea
Yesterday, they canoed down the _______.	River (0.81)	Amazon (0.01)	Woods
The ship disappeared into the thick _____.	Fog (0.89)	Mist (0.10)	Cat
At night, the old woman locked the _____.	Doors (0.94)	House (0.03)	Feast
Her job was easy most of the __________.	Time (0.99)	Way (0.01)	Hair

**Table 2:** Means and standard deviations for lexical variables of final words.

	High	Low	Anomalous	
Length (# Characters)	4.81 (1.15)	5.40 (1.59)	4.79 (1.23)	*F*(2,216) = 11.47, *p* < 0.001
Length (# Syllables)	1.32 (0.59)	1.52 (0.70)	1.35 (0.61)	*F*(2,216) = 3.33, *p* < 0.05
BNC Frequency	113.1 (189.8)	105.3 (155.5)	90.7 (146.7)	*F*(2,216) = 0.55, *p* = 0.58
KF Written Frequency	123.9 (205.2)	119.1 (186.3)	106.1 (180.3)	*F*(2,216) = 0.26, *p* = 0.77
Thorndike-Lorge WF	855.6 (1266.9)	765.4 (1138.6)	761.5 (1281.6)	*F*(2,216) = 0.19, *p* = 0.83
Brown Verbal Frequency	21.2 (49.2)	27.2 (78.9)	14.0 (36.7)	*F*(2,216) = 1.53, *p* = 0.22
Concreteness	395.7 (242.3)	379.6 (241.6)	444.2 (234.0)	*F*(2,216) = 2.67, *p* = 0.07
Imagability	411.7 (245.1)	406.2 (243.5)	455.6 (230.4)	*F*(2,216) = 1.80, *p* = 0.17
Neighborhood Size	19.1(14.0)	14.0(13.7)	18.0(13.9)	*F*(2,214) = 4.72, *p* = 0.01
Neighborhood Frequency	183.7(510.8)	104.8(300.7)	114.5(204.0)	*F*(2,212) = 1.93, *p* = 0.24

**Table 3:** Results of the Linear Mixed Effects Model.

Fixed Effects	Estimate	Std. Error	DF	*t*-Value	*p*-Value
Intercept	1388.75	55.59	121	24.98	<0.001
Continuation	−30.71	29.00	303	−1.06	0.29
Constraint	23.01	35.70	264	0.64	0.52
Group	−215.27	72.82	96	−2.96	0.004
Continuation × Constraint	−144.38	35.56	5166	−4.06	<0.001
Continuation × Group	−110.38	39.01	282	−2.83	0.005
Constraint × Group	−37.42	34.74	5077	−1.08	0.28
Continuation × Constraint × Group	110.56	48.00	5070	2.30	0.02

**Table 4:** Results of paired comparisons.

Linear Hypotheses	Estimate	Std. Error	*z*-Value	*p*-Value	*Cohen’s D*
Low-Constraint Items					
1. Control-Low Cont. vs. Control-High Cont.	−140.20	28.20	−4.92	<0.001	0.584
2. Control-Low Cont. vs. Dyslexia-Low Cont.	−216.25	73.79	−2.93	0.046	0.615
3. Control-High Cont. vs. Dyslexia-High Cont.	−326.68	73.75	−4.43	<0.001	0.968
4. Dyslexia-Low Cont. vs. Dyslexia-High Cont.	−29.76	29.45	−1.01	0.955	0.035
High-Constraint Items					
5. Control-Low Cont. vs. Control-High Cont.	−175.31	25.65	−6.84	<0.001	1.15
6. Control-Low Cont. vs. Dyslexia-Low Cont.	−252.81	72.24	−3.50	0.007	0.753
7. Control-High Cont. vs. Dyslexia-High Cont.	−253.89	66.29	−3.83	0.002	0.825
8. Dyslexia-Low Cont. vs. Dyslexia-High Cont.	−174.23	26.46	−6.59	<0.001	1.06
Additional Contrasts					
Controls					
9. Low-Cont./Low-Const. vs. Low-Cont./High-Const.	−13.93	34.40	−0.41	0.999	0.02
10. High-Cont./Low Const. vs. High-Cont./High-Const.	126.27	35.77	3.53	0.007	0.543
Dyslexia					
11. Low-Cont./Low-Const. vs. Low-Cont./High-Const.	22.64	38.04	0.60	0.998	0.028
12. High-Cont./Low-Const. vs. High-Cont./High-Const.	−121.83	37.46	−3.25	0.017	0.747

## Data Availability

The data presented in this study are available on request from the corresponding author.

## Supplementary Materials

The following are available online at https://www.mdpi.com/2076-3425/11/1/59/s1.
