# Supplementary material for: Are Linguistic Prediction Deficits Characteristic of Adults with Dyslexia?
Source: Brain Sci. 2021 Jan 6;11(1):59. doi: 10.3390/brainsci11010059 (PMC7825117; doi:10.3390/brainsci11010059)
Supplement: Supplementary file 1 [file brainsci-11-00059-s001.pdf]

# Supplementary Materials

## Section A

**Results of linear mixed effects model, including the R code and output.**

**paul_model2<-lmer(RT ~ continuation*constraint*group+(1+continuation|subject) + (1|item), data=paul, REML=FALSE)**

**summary(paul_model2)**

```
Linear mixed model fit by maximum likelihood . t-tests use Satterthwaite's method ['lmerModLmerTest']
Formula: RT ~ continuation * constraint * group + (1 + continuation |      subject) + (1 | item)
   Data: paul

     AIC      BIC   logLik deviance df.resid 
 80379.6  80465.2 -40176.8  80353.6     5318 

Scaled residuals: 
    Min      1Q  Median      3Q     Max 
-2.8134 -0.5453 -0.1447  0.3257  8.2457 

Random effects:
 Groups   Name          Variance Std.Dev. Corr 
 item     (Intercept)    15992   126.46        
 subject  (Intercept)    97292   311.92        
          continuation1   4891    69.94   -0.32
 Residual               187853   433.42        
Number of obs: 5331, groups:  item, 104; subject, 84

Fixed effects:
                                Estimate Std. Error      df t value Pr(>|t|)    
(Intercept)                      1388.75      55.59  121.34  24.982  < 2e-16 ***
continuation1                     -30.71      29.00  303.36  -1.059  0.29035    
constraint1                        23.01      35.70  264.23   0.644  0.51981    
group1                           -215.27      72.82   96.14  -2.956  0.00392 ** 
continuation1:constraint1        -144.38      35.56 5166.28  -4.060 4.97e-05 ***
continuation1:group1             -110.38      39.01  281.76  -2.829  0.00500 ** 
constraint1:group1                -37.42      34.74 5077.33  -1.077  0.28146    
continuation1:constraint1:group1  110.56      48.00 5069.86   2.304  0.02129 *  
---
Signif. codes:  0 '***' 0.001 '**' 0.01 '*' 0.05 '.' 0.1 ' ' 1

Correlation of Fixed Effects:
            (Intr) cntnt1 cnstr1 group1 cntntn1:c1 cntntn1:g1 cns1:1
continuatn1 -0.335                                                   
constraint1 -0.360  0.354                                            
group1      -0.671  0.242  0.132                                     
cntntn1:cn1  0.185 -0.688 -0.520 -0.132                              
cntntn1:gr1  0.235 -0.688 -0.245 -0.350  0.475                       
cnstrnt1:g1  0.177 -0.340 -0.498 -0.263  0.501      0.491            
cntntn1:1:1 -0.128  0.474  0.359  0.190 -0.691     -0.688     -0.724 
convergence code: 0
```

```
         Simultaneous Tests for General Linear Hypotheses

Multiple Comparisons of Means: Tukey Contrasts


Fit: lmer(formula = RT ~ tw + (1 + continuation * constraint | subject) +
    (1 + group | item), data = paul, REML = FALSE)

Linear Hypotheses:
                   Estimate Std. Error z value Pr(>|z|)    
1.0.0 - 0.0.0 == 0   -29.76      29.45  -1.010  0.95535    
0.1.0 - 0.0.0 == 0    22.64      38.04   0.595  0.99799    
1.1.0 - 0.0.0 == 0  -151.59      39.54  -3.834  0.00216 ** 
0.0.1 - 0.0.0 == 0  -216.25      73.79  -2.930  0.04630 *  
1.0.1 - 0.0.0 == 0  -356.44      74.16  -4.806  < 0.001 ***
0.1.1 - 0.0.0 == 0  -230.17      77.29  -2.978  0.04034 *  
1.1.1 - 0.0.0 == 0  -405.48      74.71  -5.427  < 0.001 ***
0.1.0 - 1.0.0 == 0    52.40      39.32   1.332  0.83435    
1.1.0 - 1.0.0 == 0  -121.83      37.46  -3.252  0.01749 *  
0.0.1 - 1.0.0 == 0  -186.49      74.11  -2.516  0.13858    
1.0.1 - 1.0.0 == 0  -326.68      73.75  -4.429  < 0.001 ***
0.1.1 - 1.0.0 == 0  -200.41      77.34  -2.591  0.11557    
1.1.1 - 1.0.0 == 0  -375.72      74.59  -5.037  < 0.001 ***
1.1.0 - 0.1.0 == 0  -174.23      26.46  -6.585  < 0.001 ***
0.0.1 - 0.1.0 == 0  -238.88      77.02  -3.102  0.02785 *  
1.0.1 - 0.1.0 == 0  -379.08      77.09  -4.917  < 0.001 ***
0.1.1 - 0.1.0 == 0  -252.81      72.24  -3.500  0.00744 ** 
1.1.1 - 0.1.0 == 0  -428.12      69.67  -6.145  < 0.001 ***
0.0.1 - 1.1.0 == 0   -64.66      74.33  -0.870  0.98027    
1.0.1 - 1.1.0 == 0  -204.85      74.24  -2.759  0.07515 .  
0.1.1 - 1.1.0 == 0   -78.58      69.56  -1.130  0.92159    
1.1.1 - 1.1.0 == 0  -253.89      66.29  -3.830  0.00220 ** 
1.0.1 - 0.0.1 == 0  -140.20      28.51  -4.918  < 0.001 ***
0.1.1 - 0.0.1 == 0   -13.93      34.40  -0.405  0.99984    
1.1.1 - 0.0.1 == 0  -189.24      35.85  -5.279  < 0.001 ***
0.1.1 - 1.0.1 == 0   126.27      35.77   3.530  0.00674 ** 
1.1.1 - 1.0.1 == 0   -49.04      33.76  -1.453  0.76583    
1.1.1 - 0.1.1 == 0  -175.31      25.65  -6.835  < 0.001 ***
---
Signif. codes:  0 '***' 0.001 '**' 0.01 '*' 0.05 '.' 0.1 ' ' 1
(Adjusted p values reported -- single-step method)
```

**Continuation (low = 0, high = 1)**

**Constraint (low = 0, high = 1)**

**Group (Control = 1, dyslexia = 0)**

**Section B**

Scatterplot showing the line of best fit. The x-axis has items based on cloze probability and the y-axis shows mean reaction time. The biggest difference between the groups is the results from the low continuation condition. For controls, there is clear separation between the high- and low-continuations across the full range of cloze probability. For dyslexics, there is no separation between high- and low-continuation at the low end of cloze probability. However, for both groups, the low-continuation condition ends up being no different from the anomalous condition.

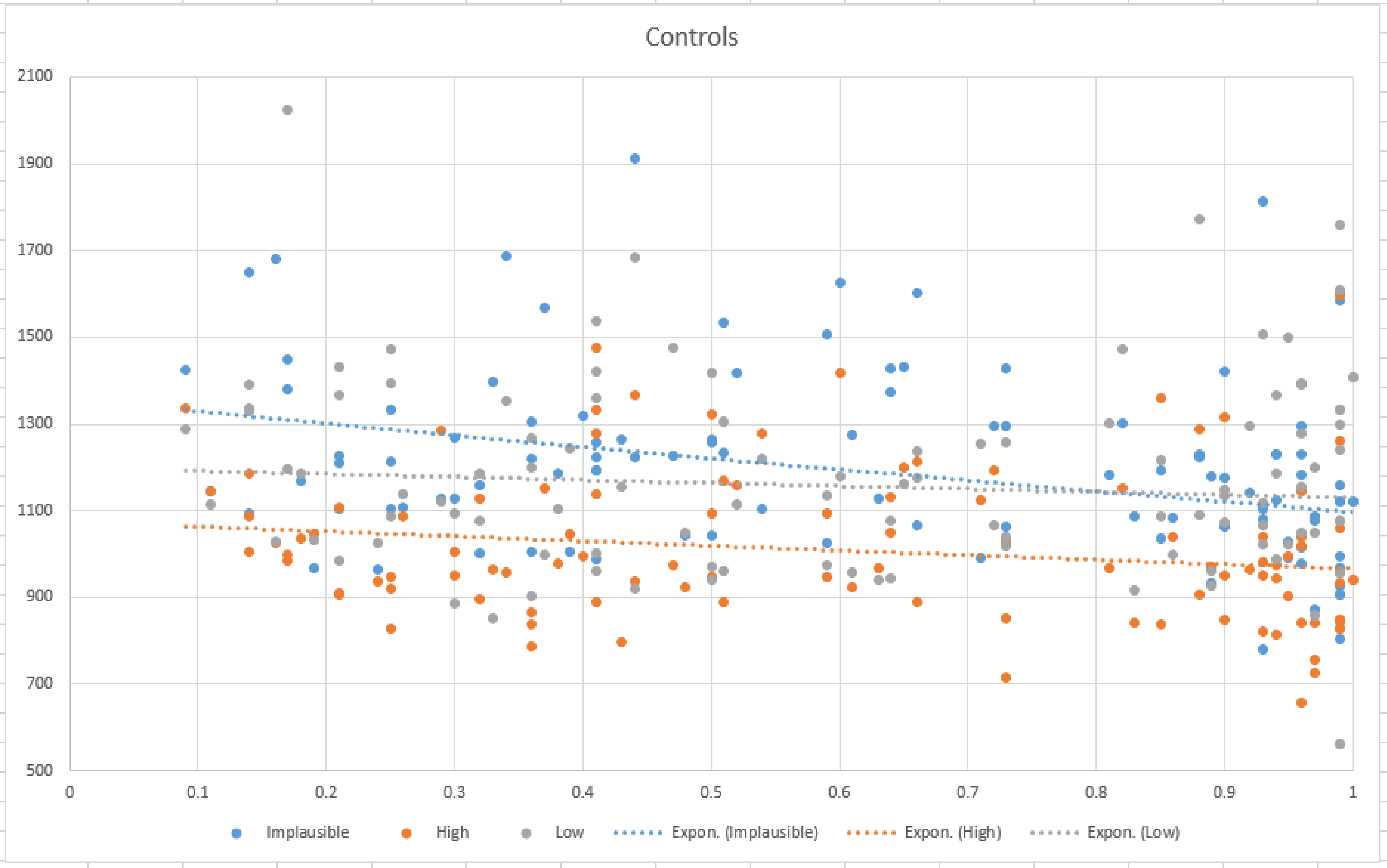

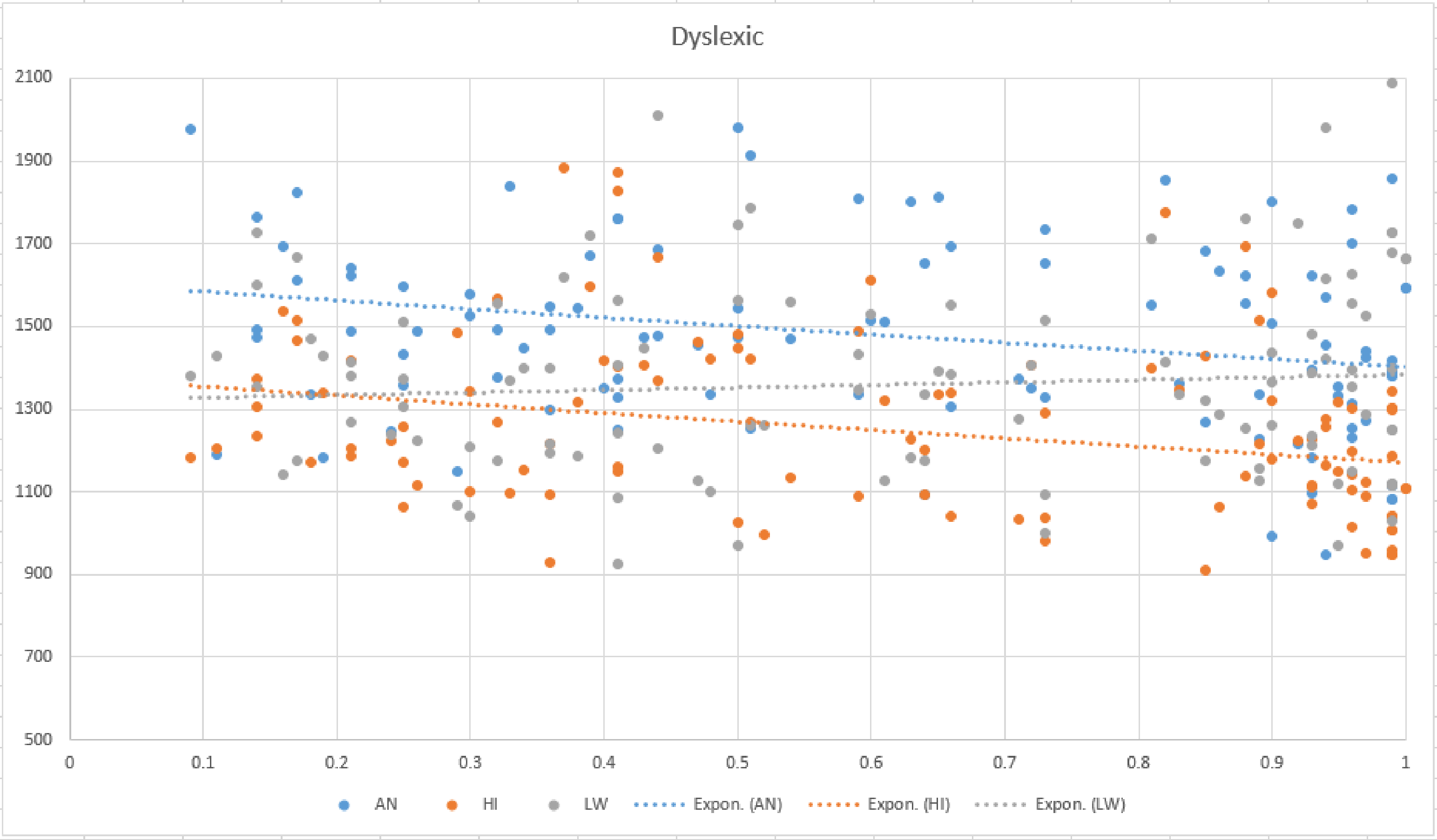
Dyslexic
2100
1900
1700
1500
1300
1100
900
700
500
0
0.1
0.2
0.3
0.4
0.5
0.6
0.7
0.8
0.9
1
AN
HI
LW
Expon. (AN)
Expon. (HI)
Expon. (LW)
